# The Potential Role of Lysosomal Sequestration in Sunitinib Resistance of Renal Cell Cancer

**DOI:** 10.15586/jkcvhl.2015.44

**Published:** 2015-01-21

**Authors:** Kaamar Azijli, Kristy J. Gotink, Henk M.W. Verheul

**Affiliations:** Department of Medical Oncology, VU University Medical Center, Amsterdam, The Netherlands.

## Abstract

Renal cell carcinoma (RCC) is a highly vascularized tumor type, which is often associated with inactivated mutations in the von Hippel-Lindau gene that drives proangiogenic signaling pathways. As such, new therapies for the treatment of RCC have largely been focused on blocking angiogenesis. Sunitinib, an antiangiogenic tyrosine kinase inhibitor, is the most frequently used first-line drug for the treatment of RCC. Although treatment with sunitinib improves patient outcome considerably, acquired resistance will emerge in all cases. The molecular mechanisms of resistance to sunitinib are poorly understood, but in the past decade, several of these have been proposed. Lysosomal sequestration of sunitinib was reported as a potential resistance mechanism to sunitinib. In this review, the underlying molecular mechanisms of lysosomal sunitinib sequestration and the potential strategies to overcome this resistance are discussed to be able to further improve the treatment of RCC.

## Introduction

Kidney cancer is one of the fastest growing cancers worldwide. Currently, it is the ninth most common cancer type in men and the 14th most common cancer type in women, with approximately 214,000 and 124,000 patients, respectively. Incidence rates are higher in developed countries wherein up to half of the cases are discovered by chance (1). The most common renal RCC subtypes are clear cell (75%), papillary (15%), chromophobic (5%), and collecting duct carcinoma (2%) (2). In addition to the four main types of RCC, there are rare, ever expanding RCC subtypes that do not fit in any of these mentioned categories.

In general, RCC is highly resistant to traditional cancer treatments, such as radiation therapy and chemotherapy (3). A better biological understanding of RCC has resulted in a rational development of targeted therapies, such as antiangiogenic tyrosine kinase inhibitors (TKIs). Currently, TKIs, such as sunitinib, pazopanib, and axitinib, are approved by the Food and Drug Administration (FDA) for the treatment of RCC. Despite the clinical benefits, prolonged progression-free survival, and overall survival of sunitinib, patients develop resistance to sunitinib and eventually experience relapse (4). Several resistance mechanisms including upregulation of proangiogenic signaling pathways, increased AXL and MET expression (5), inadequate target inhibition, and resistance mediated by the tumor microenvironment or by the action of microRNAs have been reported. Recently, a new potential resistance mechanism to sunitinib, namely lysosomal sequestration, has been reported (6). In this review, this resistance mechanism and the approaches to overcome resistance to sunitinib by using this novel knowledge are discussed.

## Angiogenesis in RCC

In RCC, which is one of the most vascularized tumors, the von Hippel-Lindau (VHL) tumor suppressor gene is inactivated in 50–75% of the cases through mutations, hypermethylations, or loss of heterozygosity (7). As a consequence of the production of pVHL, the functional protein of the VHL gene is inhibited or decreased. pVHL plays a crucial role in the downregulation of the hypoxia-inducible factor 1 (HIF1) transcription factor, which subsequently decreases angiogenesis. Upon pVHL downregulation, HIF1 is accumulated, and an increase in the transcription of HIF1 target genes, such as vascular endothelial growth factor (VEGF) and platelet-derived growth factor (PDGF), is released. Receptors for VEGF (VEGFR) and PDGF (PDGFR) are key players in angiogenesis. VEGF mediates VEGFR regulation of vessel permeability, endothelial cell activation, survival, proliferation, invasion, and migration (8). For the maintenance and stabilization of newly formed vessels, VEGF alone is not sufficient, and it requires support from the surrounding periendothelial cells, such as vascular smooth muscles (VSMCs) and pericytes. The secretion of PDGF-B by the endothelial cells and the receptor tyrosine kinases of PDGF-B located on VSMC and pericytes are involved in this crosstalk with VEGFR. The frequent inactivation of VHL provided a rationale for the development of antiangiogenic drugs, such as sunitinib, for the treatment of RCC, which will be discussed in the next paragraph.

## Sunitinib

Sunitinib (SU11248) is an oral multitargeted TKI that was granted accelerated FDA approval for the treatment of RCC and imatinib-resistant gastrointestinal tumor in 2006, being the first TKI approved for two different indications at the same time. In 2011, sunitinib was also approved by the FDA for the treatment of advanced pancreatic neuroendocrine tumors (9).

Initially, sunitinib was developed as an antiangiogenic agent inhibiting VEGFR2 and PDGFR, the two major targets, expressed in endothelial cells and perivascular cells (pericytes). Later, sunitinib was also shown to inhibit KIT, FLT3, and RET kinases and many other kinases localized in tumor cells, resulting in antiproliferative and/or apoptotic effects of these cells (10). In addition, sunitinib has no preference for tyrosine or serine-threonine kinases, indicating its broad kinase inhibition profile (10, 11). Its direct antitumor activity may also be explained by the significant accumulation in tumor tissues at clinically relevant concentrations (6), despite 10-fold lower plasma concentrations.

The clinical development of sunitinib for RCC was based on the encouraging data from the phase I trial, in which three of the four patients with RCC showed objective responses. Subsequently, a phase II trial was initiated for sunitinib, investigating its use as a second-line treatment for patients with cytokine-refractory metastatic RCC. In this phase II trial, 69 patients were enrolled and partial responses were observed in 40% and stable disease in 27% of patients. The overall median time to progression was 8.7 months (12). Because of these exceptional findings for a treatment-refractory disease as RCC at that time, a second phase II trial, in which 106 patients were enrolled, was initiated to confirm these outcomes.

Based on the findings of these two phase II studies, sunitinib received accelerated FDA approval in 2006. In both the phase II trials, the objective response rate (ORR) for sunitinib as a second-line therapy was higher than that for cytokine therapy as a first-line treatment. Given the fact that the ORR for the first-line cytokine therapy is approximately only 15% and that no drug as a second-line treatment was able to show benefit for RCC patients in clinical trials, a phase III trial was initiated to investigate the use of sunitinib in the first-line setting. In total, 750 patients worldwide were included in this trial comparing sunitinib and interferon-α (IFNα) with each other. For sunitinib, ORR was 31%, whereas for IFNα this was only 6%. The median PFS was 11 months for sunitinib versus 5 months for IFNα (13). After finishing this phase III trial, the FDA completed the approval of sunitinib in 2007 and included its use in the first-line setting for the treatment of RCC.

## Resistance mechanisms

Despite the clinical benefits achieved, patients with cancer may be intrinsically resistant or may acquire resistance to treatment with sunitinib. Approximately 70% of patients show clinical benefit to sunitinib but develop acquired resistance in 6–15 months, while 30% are intrinsically resistant (14). Understanding the molecular mechanism underlying intrinsic and acquired resistance to sunitinib may provide clues on how to circumvent this clinical problem. Several sunitinib resistance mechanisms, such as the upregulation of proangiogenic signaling pathways, increased tumor invasiveness and metastasis, activation of alternative signaling pathways, inadequate target inhibition, and resistance mediated by the tumor microenvironment or by the action of microRNAs, have been reported [for an extensive review, see the article by Joosten et al. (15)]. The evidence of most of these mechanisms has been derived from preclinical models, and their clinical relevance needs to be proven.

One factor that seems to be very crucial in sunitinib resistance is tumor hypoxia. Inhibiting angiogenesis with VEGF-targeted agents not only results in stabilization or regression of the tumor but also renders tumor cells hypoxic, leading to HIF1 accumulation (14). Subsequently, this causes upregulation of proangiogenic factors, such as VEGF that stimulates angiogenesis, cMET upregulation that increases tumor invasiveness and epithelial-to-mesenchymal transition, and stromal cell-derived factor-1 upregulation that recruits proangiogenic bone marrow-derived cells.

A recent study showed that chronic sunitinib treatment induced the activation of AXL and MET signaling and subsequently even promoted the prometastatic behavior of renal cancer cells and increased angiogenesis in a xenograft 786-O mouse model (5).

Besides the restoration of angiogenesis through the activation of alternative pathways, reduced bioavailability through increased efflux by drug pumps such as the ATP-binding cassette (ABC) superfamily or lysosomal sequestration leading to inadequate target inhibition may be another factor contributing to resistance of sunitinib. In the next paragraph, the lysosomal sunitinib sequestration is discussed in more detail.

## Lysosomal sequestration

Lysosomes are acidic intracellular organelles containing acidic hydrolases capable of degrading biological macromolecules, such as nucleic acids, lipids, and proteins. In addition, lysosomes are involved in recycling defective organelles, exocytosis, apoptosis, and autophagy. Hydrophobic weak base chemotherapeutic drugs, such as doxorubicin, daunorubicin, mitoxantrone, and imidazoacridinones, have been shown to accumulate in lysosomes. Recently, this was also demonstrated for sunitinib in renal and colon cancer cells, after the observation was made that the intracellular sunitinib concentration was 10-fold higher in resistant cells than in sensitive cells, providing a new resistance mechanism for this TKI (6). The hydrophobic properties of sunitinib (log P = 5.2) allow the drug to cross cell membranes easily via passive diffusion. However, because sunitinib is a weak base (pKa = 8.95), it becomes protonated in an acidic environment and loses its ability to cross membranes. Therefore, upon entering into lysosomes, sunitinib is entrapped in its cationic state in these acidic organelles. Remarkably, despite the increased intracellular sunitinib concentration in resistant cells, kinase activity was unaffected. p-Akt and p-ERK levels in resistant cells were similar to the levels in untreated parent cells. Increased sequestration of sunitinib in lysosomes of resistant tumors has also been demonstrated in in vivo experiments. The expression of lysosomal-associated membrane protein (LAMP)-1 and -2, which reflects lysosomal capacity, was found to be higher in sunitinib-resistant tumors when compared with that in parental tumors (16). Lysosomal sequestration as a resistance mechanism was also shown for other TKIs, such as gefitinib and lapatinib in immortalized human hepatocytes (Fa2N-4 cells) (17). In addition, pazopanib and erlotinib showed increased intracellular accumulation measured with liquid chromatography-tandem mass spectrometry system and an elevated expression of LAMP-1 and LAMP-2 in resistant renal (786-O) and colorectal cancer cells (HT29), suggesting an involvement of the lysosomal compartment (18). These compounds have the same chemical properties as sunitinib, being hydrophobic weak base TKIs (**Table 1**). Although lysosomal sequestration of sorafenib was not found in renal and colorectal cancer cells (17), it has been demonstrated in hepatocellular carcinoma (HCC) by Colombo et al. (19). Sorafenib does not belong to the same class of hydrophobic, membrane-permeable weak base as sunitinib, and therefore, a different mechanism could explain its lysosomal sequestration, probably an active involvement of drug pumps.

**Table 1.** Lysosomal sequestration of TKIs in several different cell lines

Drug	Calculated log P	Calculated pKa (strongest basic)	Lysosomal sequestration found in	References
Sunitinib	5.2	8.95	Renal and colon cancer cells	(6)
Gefitinib	3.2	7.20	Hepatocytes	(17)
Lapatinib	5.4	7.20	Hepatocytes	(17)
Pazopanib	3.6	5.07	Renal and colon cancer cells	(18)
Erlotinib	3.2	4.59	Renal and colon cancer cells	(18)
Sorafenib	4.34	2.03	Hepatocellular carcinoma	(19)

**Figure 1.**
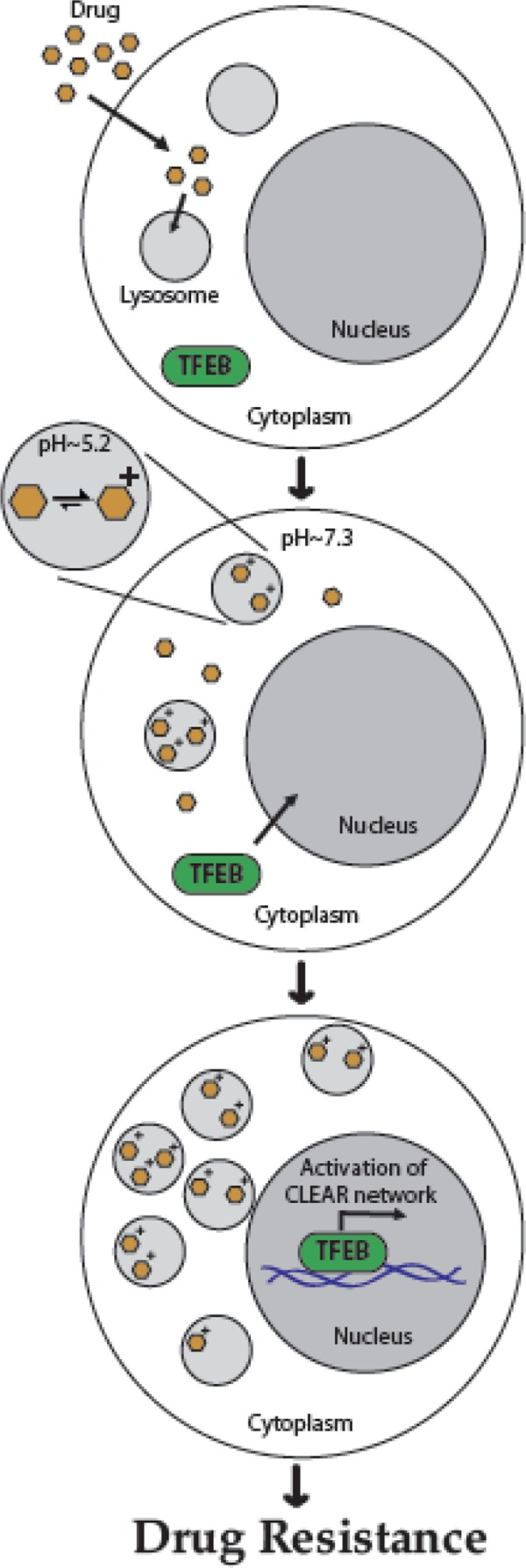
Schematic summary model for hydrophobic weak base drug-induced lysosome-mediated drug resistance. Hydrophobic weak base drugs enter the lysosomes by simple diffusion and undergo protonation in the acidic lysosomal lumen, thereby becoming irreversibly sequestered in lysosomes and acidic intracellular vesicles such as late endosomes. In turn, lysosomal drug sequestration triggers TFEB-mediated lysosomal biogenesis, resulting in a significant increase in the number of lysosomes per cell. Increased lysosome number per cell enhances the efficiency of lysosomal drug sequestration, with lysosomes acting as a sink pulling hydrophobic weak base drugs away from their cellular target sites, thereby resulting in MDR (21).

The lysosomal sequestration of sunitinib and sorafenib was reported to be mediated by the ABC transporter P-glycoprotein (Pgp) (19). This drug pump actively effluxes various cytotoxic compounds from the cells for cytoprotection. Pgp expression was found not only in cell membranes but also in lysosomes, being involved in actively sequestering sunitinib into these organelles. On the surface of lysosomes, the transcription factor EB (TFEB) forms a complex with mammalian target of rapamycin (mTOR1) (mTORC1). When TFEB becomes phosphorylated at Ser211 by mTORC1, it interacts with 14-3-3 and remains in the cytoplasm. However, as a consequence of aberrant lysosomal storage, cell starvation, or mTORC1 inhibition, TFEB dissociates from the lysosome and translocates to the nucleus and increases the expression of genes encoding lysosomal proteins (20). Recently, it was found that lysosomal sequestration of hydrophobic weak base chemotherapeutics, including sunitinib, triggers TFEB-mediated lysosomal biogenesis, resulting in a significant increase in the number of lysosomes per cell. As a consequence, the efficiency of lysosomal drug sequestration and therefore multidrug resistance increases even further (21) (see **Figure 1**).

## Overcoming sunitinib resistance by disturbing lysosomal sequestration

Lysosomal sequestration seems to eliminate the cytotoxic effect of sunitinib by decreasing drug concentrations at the intracellular target site. Therefore, a potential approach to overcome resistance to sunitinib may be combination treatment with drugs that circumvent lysosomal drug sequestration. A better understanding of the mechanisms underlying lysosome-mediated drug resistance, which have been discussed in the previous paragraph, is therefore of great importance. The extent of lysosomal drug sequestration has been shown to depend on the pH gradient between the acidic luminal pH of the lysosome and that of the cytoplasm (22). In this respect, lysosomal drug accumulation can be reversed with agents that alkalinize lysosomes, such as bafilomycin A1, a H+-ATPase inhibitor. In in vitro experiments, this compound was shown to reverse lysosomal sunitinib sequestration. However, this compound is too toxic for in vivo treatment. Therefore, in mice, chloroquine, which inhibits lysosomal function by raising lysosomal pH, was used (16). Currently, chloroquine and hydroxychloroquine are the only clinically available inhibitors of autophagy.

Despite the findings and the potential mechanism of resistance as described above, concanamycin A, a vacuolar-type H^+^-ATPase inhibitor, reduced the amount of cell death induced by sunitinib in breast cancer MCF7 cells dramatically due to relocalization of sunitinib into the cytosol (23). This suggests that lysosomal sequestration seems to be essential for the cytotoxic activity of sunitinib. The discrepancy that lysosomal sequestration explains sunitinib resistance on one hand and is important for antitumor activity of sunitinib on the other hand could be cell-type specific. This also indicates that resistance to an antitumor agent is a complex process and involves several different molecular mechanisms.

**Figure 2.**
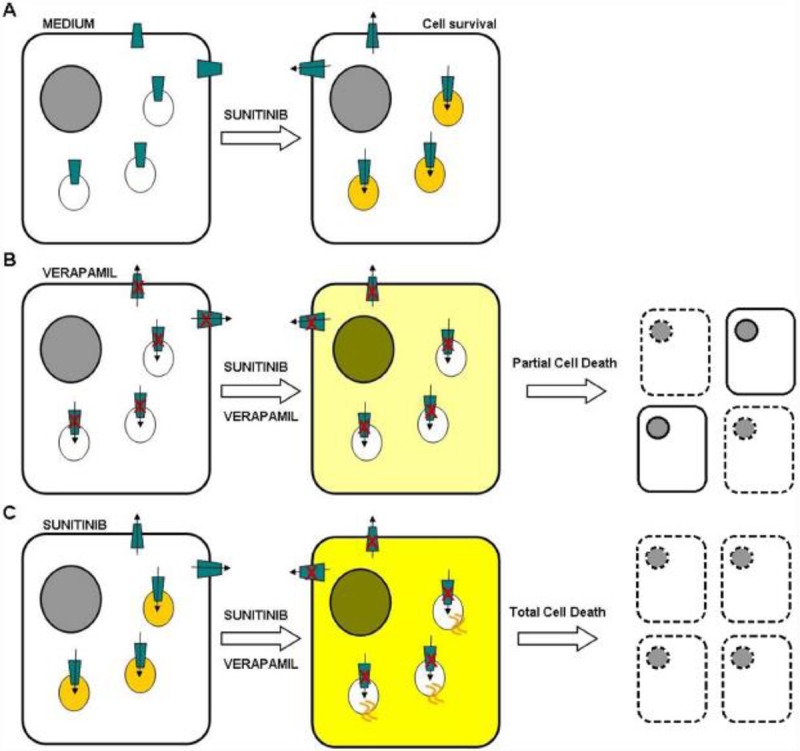
Hypothesized mechanism of the enhanced efficacy of drug pretreatment before verapamil administration and PGP blockade. **A.** HCC cells expressing active PGP can expel a drug (e.g., sunitinib) from the cytoplasm or store it in lysosomes. **B.** Blocking PGP with verapamil before the coadministration of sunitinib and verapamil allows the drugs to enter the cell and diffuse into cytoplasm/nucleus. **C.** If sunitinib is used for pretreatment, it is stored in giant lysosomes, and after the coadministration of sunitinib and verapamil and subsequent PGP blockade, the drugs can enter the cytoplasm/nucleus from both extracellular space and the lysosomes (19).

Alternative data showing that lysosomal accumulation of sunitinib is involved in resistance are provided by the reports showing that the overexpression of Pgp in lysosomes enhances intralysosomal drug sequestration. Subsequently, inhibition of this drug pump with verapamil restored sensitivity to TKIs, including sunitinib, especially when administrated after drug preincubation. An explanation that during the preincubation phase, anticancer drugs are being trapped in Pgp-positive lysosomes was given by the authors. Blocking Pgp activity by subsequent incubation with the drug/verapamil combination allows drug diffusion from the culture medium and lysosome into the cytoplasm (**Figure 2**). As a consequence, the intracellular drug concentration is increased (19). The clinical use of verapamil is limited due to its cardiac toxicity. Alternatively, due to the fact that verapamil undergoes extensive hepatic first-pass metabolism, it is theoretically possible to avoid this side effect by using intrahepatic injections in combination with chemoembolization (24, 19).

Switching to another TKI when resistance to sunitinib occurs is a practical clinical strategy to consider. However, this option is not always feasible and often does not solve the problem due to cross-resistance. For most of the antiangiogenic TKIs, including pazopanib, erlotinib, and lapatinib, cross-resistance in sunitinib-resistant RCC cells was found (18). The antitumor activity of sorafenib and the mTOR inhibitor, everolimus, did not decrease upon sunitinib resistance. Switching to these drugs is therefore a potential option when patients with RCC are resistant to sunitinib treatment.

Sequential therapy with everolimus is preferred over combining this drug with sunitinib. As described in the previous paragraph, lysosomal sunitinib sequestration increases via TFEB-mediated lysosomal biogenesis (21). This can be achieved through mTOR inhibition. In addition, the combination of sunitinib with everolimus was associated with significant toxicities (25). An intriguing observation made in the laboratory is that when exposed to light, sequestered sunitinib caused immediate destruction of the lysosomes, resulting in the release of sunitinib and cell death. Although combining sunitinib with phototherapy could therefore be an interesting approach to overcome sunitinib resistance caused by lysosomal sequestration (26), its practical use is very limited due to the superficial and local treatment options with phototherapy. A more practical approach for patients with metastatic disease requiring systemic exposure is urgently needed. Several interesting combination therapies to overcome sunitinib resistance in metastatic renal cancer are being explored in preclinical and clinical studies (27). It is of high interest to see the outcome of the phase I trial (NCT00813423) in which sunitinib is combined with hydroxychloroquine in patients with advanced solid tumors that have not responded to chemotherapy to better understand whether disturbing lysosomal sunitinib sequestration is clinically involved in its resistance.

## Conclusions

Sunitinib is a very active first-line drug for the treatment of RCC. However, due to the chemical properties of sunitinib, this compound becomes sequestered in lysosomes, preventing the drug from reaching its target. After being treated for a period of time, most patients with RCC develop resistance to sunitinib potentially as a consequence of drug accumulation in lysosomes. Some studies have investigated the molecular mechanism of this novel resistance mechanism in more detail, providing clues for the concomitant treatment of sunitinib with drugs that interfere with lysosomal function.
